# Post-Traumatic Stress Symptoms Following Childbirth: A Contribution to the Psychometric Evaluation of the Greek Version of the Traumatic Event Scale (TES) (Version B)

**DOI:** 10.3390/healthcare13070768

**Published:** 2025-03-30

**Authors:** Pinelopi Varela, Ioannis Zervas, Athina Diamanti, Christina Nanou, Aikaterini Lykeridou, Anna Deltsidou

**Affiliations:** 1 General Hospital of Athens “Alexandra”, 11528 Athens, Greece; 2 Department of Midwifery, University of West Attica, 12243 Athens, Greece; adiamanti@uniwa.gr (A.D.); nanouxv@uniwa.gr (C.N.); klyker@uniwa.gr (A.L.); adeltsidou@uniwa.gr (A.D.); 3 First Department of Psychiatry, School of Medicine, National and Kapodistrian University of Athens, Eginition Hospital, 11528 Athens, Greece; izervas@med.uoa.gr

**Keywords:** post-traumatic stress symptoms, post-traumatic stress disorder, PTSD, postpartum, Traumatic Event Scale, psychometric properties, validity, reliability, Greece

## Abstract

**Background/Objectives**: Research has shown that postnatal post-traumatic stress (PTS) symptoms may adversely affect women’s lives, their infants’ development, and their relationships with their partners. The study aimed to evaluate the psychometric features of the Traumatic Event Scale (TES) (version B) in a sample of Greek postpartum women. **Methods**: Two hundred women completed the Greek version of the TES-B (GrTES-B) and the Edinburgh Postnatal Depression Scale (EPDS) at four weeks postpartum. **Results**: The GrTES-B data from the sample were applied to the previously established five-factor structure of version A of the TES (“Anticipation of trauma”, “Intrusion”, Avoidance”, “Resignation”, and “Hyperstimulation”) employing confirmatory factor analysis. Considering that every Cronbach’s alpha was greater than 0.7, the factors’ reliability proved satisfactory. Significant correlations were observed regarding the convergent and divergent validity, indicating the instrument’s sufficient validity. **Conclusions**: The Greek version of the TES-B demonstrated satisfactory psychometric characteristics for the assessment of PTS symptoms among Greek postpartum women.

## 1. Introduction

Post-traumatic stress disorder (PTSD) and post-traumatic stress (PTS) symptoms during the postpartum period are among the mental health issues after childbirth that have received the scientific community’s attention [1,2,3]. PTSD is a psychiatric disorder that may occur after exposure to death, threatened death, or serious injury, whether experienced or witnessed. The perception of “threatened death or injury” is subjective and determined by the person experiencing it. The diagnosis of PTSD requires exposure to extreme stressors or traumatic events that are accompanied by the PTS symptoms [4,5].

The fifth edition of the Diagnostic and Statistical Manual of Mental Disorders (DSM-5) includes twenty PTSD symptoms that are grouped into four clusters: (1) intrusions/reexperiencing the traumatic event, (2) avoidance of reminders of the event, (3) negative cognitions and mood, and (4) alterations in arousal and reactivity. To be diagnosed with PTSD, an individual should have several symptoms from each category present. The symptoms need to last longer than a month and cause distress and/or impairment [5,6]. In addition, some PTSD symptoms are observed in individuals with other health conditions, including anxiety and depression disorders, according to the research on PTSD [7,8]. Even though the traumatic event is already included as a cause in the PTSD diagnostic criteria, the fact that most individuals who experience trauma do not develop PTSD demonstrates that the likelihood of developing this disorder varies significantly among individuals [9,10]. Despite this, according to the literature, culture may influence individuals’ reactions to trauma by “providing meaning to symptoms, by shaping individuals’ beliefs about traumatic events, by affecting individuals’ beliefs about their own responsibility for the trauma and their subsequent response, by indicating what disabilities or impairments may result from the trauma, and by shaping the threshold for normal versus pathological levels of arousal” [11]. As a result, different cultural contexts may have diverse definitions and interpretations of traumatic events. Consequently, the cultural context should also be considered while assessing PTSD. Since for the assessment of the symptomatology of the disorder, self-report scales are commonly used [12], the cross-cultural adaptation of these is of important significance [13].

In Greece, due to a relative dearth of research data, there is no extensive information available on the prevalence rates of PTSD or PTSD symptoms. However, studies in Greece have examined different groups exposed to various potentially traumatic events [14]. Population groups that have been the focus of PTSD research in Greek settings include children, university students, and healthcare professionals. Data have also been published on the prevalence of PTSD symptoms in relation to the COVID-19 pandemic. Among the past studies involving Greek data is that of Giannopoulou et al. (2006), which explored the differences in PTSD symptoms between a group of children exposed to an earthquake and a group of children not exposed to it. Compared to 20.1% (*n* = 56) of the children in the indirect exposure group, 35.7% (*n* = 623) of the children in the exposure group scored above the cut-off point for intrusion and avoidance items in the assessment tool [15]. In 2017, Antonopoulou et al. investigated childhood trauma in 605 undergraduate and postgraduate students from Athens universities, and they found that the most frequent types of trauma were corporal punishment (89.9%), emotional abuse (67.2%), and sexual abuse (27%) [16]. Another study with a sample of university students is that of Andreou et al. (2020), who investigated the association between bullying victimization experiences at school and current PTSD symptoms among 400 Greek university students. The results showed that victims of school bullying reported mild levels of PTSD symptoms [17]. A recent study with a sample of 112 healthcare workers working with refugees showed that 17.9% (*n* = 20) had PTSD symptoms [18]. In a large web-based survey with a sample of 1661 participants, Kalaitzaki examined the psychological impact of the COVID-19 lockdown in Greece. A high percentage of the general population (27.2%) had a positive score for PTSD symptoms, and nearly all healthcare workers (99.7%) reported a moderate level of PTSD symptoms [19]. One year later, Pappa et al. (2022) published a paper in which they mentioned that 37.4% (*n* = 49) of the 133 participants with subsequent hospitalization as a result of the SARS-CoV-2 infection had PTSD symptoms [20]. Another study on the assessment of PTSD in healthcare professionals during the pandemic found that of 162 participants, 35% (*n* = 56) had a score in the assessment tool that suggested PTSD [21].

As previously mentioned, being exposed to a traumatic event is considered to be an essential requirement for the disorder’s diagnosis [4,5]. Childbirth is an event that can be experienced or perceived by women as a traumatic experience, described as “a woman’s experience of interactions and/or events directly related to childbirth that caused overwhelming distressing emotions and reactions, leading to short- and/or long-term negative impacts on a woman’s health and wellbeing” [22]. A traumatic experience of childbirth may result in the development of postpartum post-traumatic stress disorder (PTSD) or in the manifestation of postnatal post-traumatic stress (PTS) symptoms [23,24]. Bydlowski and Raoul-Duval (1978) were among the first who published their observations regarding postnatal PTSD, with a description of “la nervose traumatique post-obstetricale” (post-obstetric traumatic neurosis) among women who had experienced long and difficult labors, instrumentally assisted births, or the birth of a dead or injured infant [25]. However, what constitutes a traumatic childbirth experience depends on the woman who experienced it. Consequently, even when there was no objective risk to the mother’s or her child’s life, the woman may have felt that her labor was a traumatic event [26].

Based on past literature data, it is generally reported that between 29% and 44% of women report having a traumatic childbirth experience [27,28,29,30]. However, the recent literature suggests that an estimated one-third of women who gave birth experienced extremely stressful and possibly traumatic childbirths [31,32,33]. Furthermore, studies have shown that between 3% and 6% of mothers experience postnatal PTSD [34] and 18.5% to 41.2% of women will develop PTSD after a difficult childbirth [35,36,37]. According to estimates, between 12% and 13% of postpartum women experience subclinical PTS symptoms; in high-risk groups, such as those following a preterm birth or serious complications, this percentage rises to 16% to 19% [34,35,38].

Several risk factors for the onset and expression of postpartum PTSD have been identified. The burdened psychological state of women, such as a history of PTSD, perinatal depression, fear of childbirth, and poor coping skills, is commonly found in the literature. Obstetric interventions and complications, including preterm birth, unplanned caesarean section, instrumental delivery, obstetric emergencies, neonatal morbidity, and perinatal mortality, are among the other risk factors [38,39,40].

PTSD and PTS symptoms during the postpartum period may result in a negative impact on a mother and child’s health. Postpartum women experience a variety of symptoms, such as nightmares, sleep and concentration problems, flashbacks, negative alterations in mood and cognition, and avoidance of reminders of the trauma [41,42,43]. In addition, studies have shown that postpartum PTSD and PTS symptoms are associated with reduced breastfeeding [44], difficulties with the early development of bonding between the mother and her child [43,45,46], child sleep problems [47], requests for a cesarean delivery in later pregnancies [48], and pregnancy avoidance in the future [49].

PTSD and PTS symptoms are not regularly screened throughout pregnancy and the postpartum period and so remain mostly unrecognized in maternity services. Consequently, affected women are not recognized, and as a result, they do not receive prompt, suitable care or treatment for postpartum PTSD or its symptoms [31,50,51]. Evidence suggests that PTSD may be prevented by providing early interventions before the full onset of traumatic stress disorder [52,53,54], and it has been stated that maternal mental health screening on its own may be beneficial [55]. Therefore, screening for PTSD and its symptomatology is a crucial stage.

Among the screening tools for PTSD symptomatology that have been used in Greek studies are the Greek versions of the Early Trauma Inventory Self-Report—Short Form (ETISR-SF) [16,56], the 20-item Posttraumatic Check List-5 (PCL-5) [18,57], the Impact of Event Scale—Revised (IES-R) [58,59] and the PTSD Checklist—civilian scale (PCL-C) [60,61].

The Traumatic Event Scale (TES) is one of the psychometric scales used for the assessment of PTS symptoms; it was created specifically for evaluating PTSD symptomatology during the perinatal period [62]. To date, version B of the TES (TES-B), which concerns the evaluation of postpartum PTSD symptomatology, has not been validated in Greece, and therefore its application for the assessment of postpartum PTSD symptomatology cannot be considered valid. The aim of the present study was the examination of the psychometric properties of TES-B among Greek postpartum women. The TES-B was chosen for validation for the following reasons: (a) it was developed in compliance with the DSM-IV criteria for PTSD and contains all of the PTSD symptom criteria [62], (b) it has been employed in several studies, with a focus on women’s postpartum mental health [27,29,63,64,65] and, (c) given that the TES-A has already been validated in the Greek pregnant population, the examination of TES-B’s psychometric properties would enhance the evaluation of PTSD symptomatology throughout the perinatal period in Greece. The identification of women with the symptomatology is important given the detrimental impacts of the condition, and the existence of validated instruments is a necessary part of their identification.

## 2. Materials and Methods

### 2.1. Traumatic Event Scale Version B (TES-B)—Translation Stage

There were four steps in the translation process: forward translation, translation synthesis, back translation, Expert Committee, and documentation submission to the developer. All of the aforementioned stages were carried out with approval from Professor Klaas Wijma, the scale’s creator [62].

### 2.2. Traumatic Event Scale Version B (TES-B)—Pilot Testing Stage

The TES-B was completed at various periods by a sample group of thirty postpartum women. The completion time of the scale was 10 min on average. Test–retest reliability was evaluated using intraclass correlation coefficients (ICCs), which had a range of 0.56 to 1.00. The internal consistency was assessed using the Cronbach’s alpha (Cronbach’s a) reliability coefficient of five dimensions of the scale, which varied between 0.62 and 0.87. The detailed translation process and pilot study information have been previously published [66]. The Greek version of the TES (GrTES) Version B was developed following the findings of the pilot study.

### 2.3. Study Sample

The study’s participants were postpartum women who had given birth one month earlier. The following were the criteria for inclusion: postpartum women who were at least eighteen years old, had a low-risk pregnancy, and had an adequate understanding of Greek. Postpartum women who had a high-risk or multiple pregnancy, a severe chronic disease, a psychiatric illness, or were under psychiatric medication were not included. It was calculated that with the sample size of 200 participants, the study would have enough power (>85%) to perform a factor analysis for GrTES-B.

### 2.4. Study Procedure

The recruitment process occurred during women’s regular prenatal examination. It began by identifying the potential participants based on inclusion and exclusion criteria by thoroughly examining their medical histories. Potential participants were approached in person by the principal researcher and were asked to have a conversation with her. During this conversation, each woman received a detailed overview of the study, including its stages, its requirements, and its benefits, to ensure they were well-informed before deciding to participate. Following the aforementioned procedures, participants were invited to take part in the study. An informed consent form was signed by each participant before their involvement. The study took place at a public maternity facility in Athens between July 2020 and December 2021. 

**Figure 1:**
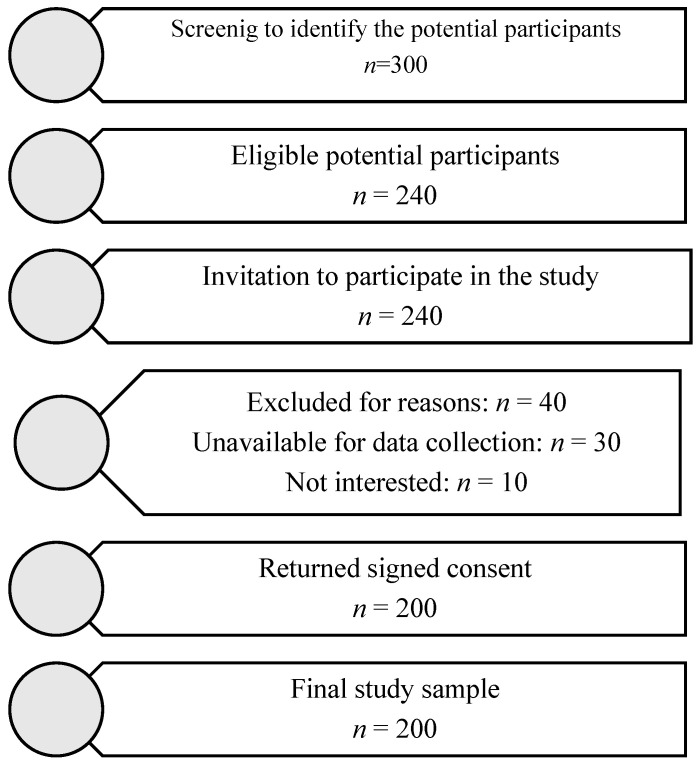
Flowchart of participant recruitment and retention.

Two hundred of the 240 women who were initially invited to take part in the study comprised the final sample. Figure 1 shows the flowchart of participant recruitment and retention. The participants were asked to complete two psychometric evaluations and a variety of questionnaires that were provided to them. Demographic data, mental health information, obstetric history, and details about the recent delivery and postpartum period were all covered in the questionnaires that were administered.

### 2.5. Measures

#### 2.5.1. Traumatic Event Scale Version B (TES-B)

The TES-B is a self-report questionnaire that was developed to measure PTS symptoms following childbirth. It includes all PTSD symptom criteria and was developed in line with the DSM-IV criteria. Criterion A consists of four statements that concern the particular trauma of interest, i.e., the traumatic childbirth experience, and each statement is followed by four possible answers: “not at all”, “somewhat”, “somehow” “much”, and “very much”. PTSD symptoms, such as reexperiencing, avoidance, and hyperarousal, are included in the 17 sentences that follow criterion A and relate to criteria B, C, and D. Respondents select one of four alternatives to rate the frequency of the symptoms listed in the statements: “never/not at all”, “rarely”, “sometimes”, or “often”. Criterion F is assessed by participants’ ratings on a scale from 0 to 10 (“not at all” to “extremely influenced”) regarding the degree to which their life is influenced by the symptoms. The duration of symptoms concerns criterion E, and it is assessed using a 13-point scale, ranging from “less than 4 weeks” to “more than 12 months”. Therefore, for the assessment of criterion E, the duration of symptoms should be at least one month. Consequently, the scale was completed by the participants four weeks after the delivery. Cronbach’s a and split-half reliability were 0.84 and 0.90, respectively, for the original version [62]. Participants were asked to complete the Greek version of the TES-B (GrTES-B).

#### 2.5.2. Edinburgh Postpartum Depression Scale (EPDS)

The EPDS consists of ten items that describe symptoms of depression. Each of the four possible responses is ranked based on the severity of the symptom. After the responses are graded from 0 to 3, the sum of their scores is determined [67]. The internal consistency reliability of the Greek version of the scale after its translation and validation is excellent (Cronbach’s a = 0.9) [68].

### 2.6. Statistical Analysis

The absolute (N) and relative (%) frequencies were used to describe the qualitative variables. Mean values (mean), standard deviations (SD), medians (median), and interquartile ranges (IQR) were utilized to describe quantitative variables. Confirmatory factor analysis (CFA) with the maximum likelihood procedure was conducted to test how well the French version of the TES-A five-factor model [69] fits the GrTES-B data. The aforementioned five-factor second-order model has already been shown to be accepted in GrTES-A data [70]. The comparative fit index (CFI), the Tucker-Lewis index (TLI), and the root mean square error of approximation (RMSEA) were used as goodness-of-fit indices [71]. These indicators were considered adequate when CFI ≥ 0.90, TLI ≥ 0.90, and RMSEA ≤ 0.05 [72,73,74]. Cronbach’s coefficient was calculated to assess internal consistency reliability. Reliability values of 0.70 or higher were considered satisfactory for the scales [75]. Spearman’s correlation coefficient (r) was used for the performance of the convergent and divergent validity for correlations between the GrTES-A and the GrTES-B. Convergent validity was tested through intercorrelations among the five TES factors, and divergent validity of the scale was assessed with the EPDS. Statistical significance was set at *p* < 0.05, and analyses were conducted using SPSS (version 26.0) and STATA (version 13.0).

## 3. Results

### 3.1. Sample Characteristics

Two hundred (*n* = 200) postpartum women, with an average age of 34.3 years (SD = 4.2), comprised the sample of the study. All participants responded to all questionnaire items, and therefore there were no missing data. The majority of the sample had Greek nationality (96.0%), were married/living with their partner (99.5%), were employed (79%), held a university degree as their level of education (64%), and had a supportive environment (93.5%). In addition, 48% had children already, and 53.2% of participants would characterize their previous childbirth experience as mainly positive. Moreover, 32.5% (n = 65) of women had previously visited a professional for psychological difficulties they were experiencing, with anxiety being the most common cause, at 46.2% (*n* = 30). Additionally, 6.5% (*n* = 13) of participants reported having previously used medication for psychological conditions. Furthermore, 24.5% (*n* = 49) had experienced abuse in childhood, and 24.5% (*n* = 49) in adulthood. Forty-one per cent of participants (*n* = 82) had experienced a traumatic or highly stressful event in their lives in the last year, with the most common being the illness of a close person (37.8%, *n* = 31) and financial difficulties (35.4%, *n* = 29). The average gestational age at delivery was 38.8 weeks (SD = 0.8), and 98% of newborns were full-term. The majority of the sample had a vaginal delivery (80%), with 53.5% of participants characterizing their childbirth experience as mainly positive. Table 1 presents the characteristics of the sample.

### 3.2. Confirmatory Factor Analysis

**Figure 2:**
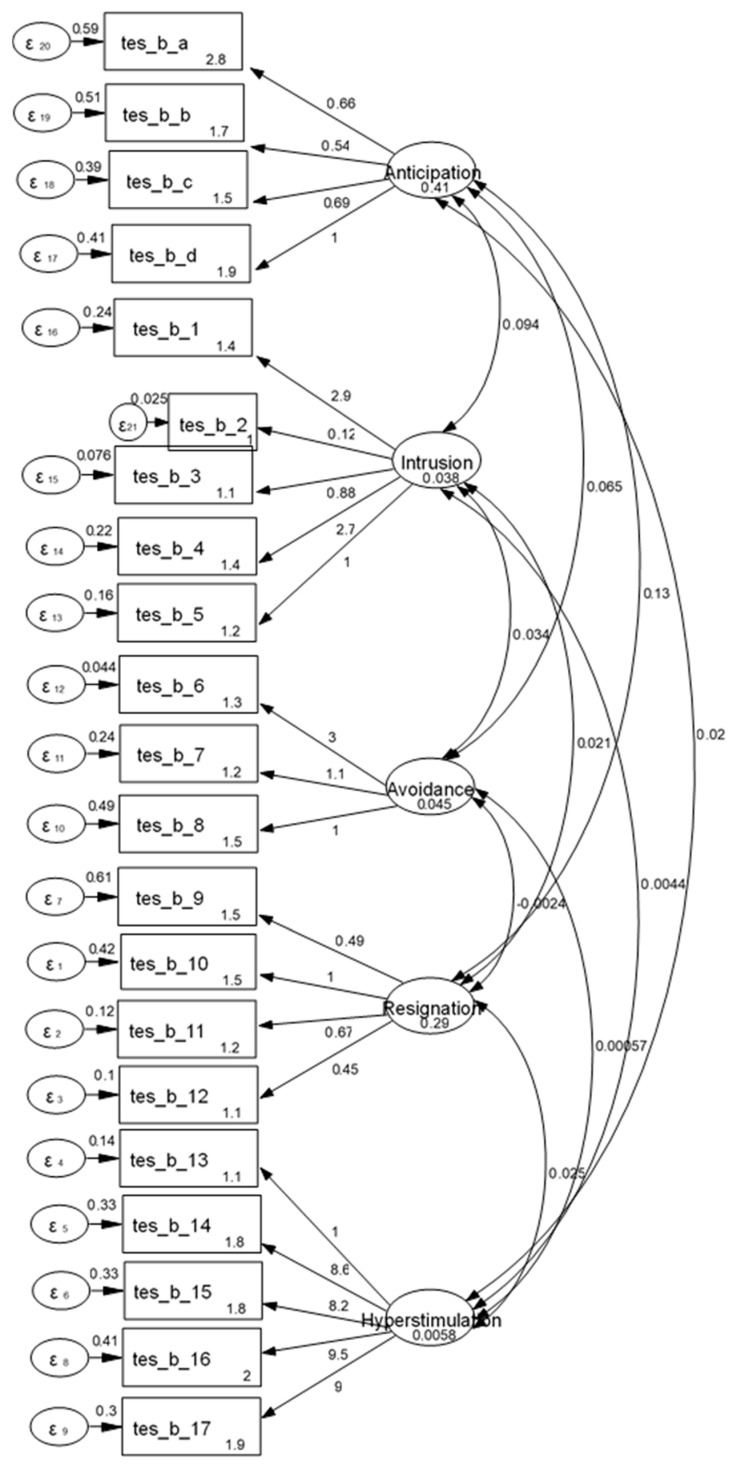
CFA results.

CFA was conducted in order to test the fitting of the French version of the TES-A five-factor model. Item loadings are presented in Figure 2. Initial CFA indexes were CFI = 0.84, TLI = 0.81, and RMSEA = 0.09. However, after applying modification indexes, CFA revealed an acceptable fit for the scale (CFI = 0.95, TLI = 0.92, and RMSEA = 0.07), and so the five-factor structure was acceptable. The first factor is named “Anticipation of trauma”, the second factor is called “Intrusion”, the third factor is named “Avoidance”, the fourth factor is called “Resignation”, and the fifth factor is named “Hyperstimulation”.

### 3.3. Descriptive Statistics on the Distribution of Responses and the Mean Scores on the GrTES-B and the Internal Consistency of the GrTES-B

Table 2 presents descriptive statistics for the GrTES-B. Table 3 presents the mean of the participants’ scores on the dimensions of the GrTES-B, item-total correlations, and Cronbach’s a for each factor. All Cronbach’s a scores were above 0.7, and the overall Cronbach’s a was 0.82, indicating acceptable reliability. There was no reason to eliminate any of the items because this would not improve the coefficients. Also, the correlation coefficients of each item with the total score of each dimension were considered satisfactory (>0.3).

### 3.4. Convergent and Divergent Validity

Convergent validity was demonstrated by the significant positive correlations found between nearly all of the GrTES-B’s dimensions. The Spearman correlation coefficients between the GrTES-B’s dimensions are displayed in Table 4.

Regarding divergent validity, nearly every factor of the GrTES-B showed a significant positive correlation with the EPDS. The “Avoidance” dimension is an exception, since it did not exhibit a significant correlation with the EPDS. Table 5 demonstrates the Spearman correlation coefficients between the dimensions of the GrTES-B and the EPDS.

### 3.5. Correlation Coefficients Between the GrTES-A and the GrTES-B

Significant positive correlations were observed between almost all factors of the two versions (A and B) of the GrTES. The exceptions are the dimensions “Avoidance” of GrTES-B and “Hyperstimulation” of GrTES-A, which were not significantly correlated with each other. Table 6 presents in detail the Spearman correlation coefficients between the dimensions of the two versions of the GrTES tool.

## 4. Discussion

The purpose of this study was to examine the psychometric properties of TES (version B) in postpartum Greek women. The TES-A’s five-factor structure, which was revealed from a prior published French study [69] was applied to our sample. Therefore, the GrTES-B, i.e., the Greek version of the TES-B, consists of the 21 items of the original version [62] and has the same five-factor structure as that identified in the French population [69]. Moreover, the five factors of the GrTES-B are the same as those of the GrTES-A [70], and their names are as follows: “Anticipation of trauma”, “Intrusion”, “Avoidance”, “Resignation”, and “Hyperstimulation”. The factors of the two versions of GrTES not only are the same but also are significantly correlated with each other in most cases. In light of everything mentioned above, there is an increased likelihood that the TES-A is not a unidimensional tool. Furthermore, the fact that the multidimensional structure of the scale concerns two different nationalities of women suggests that there are some fundamental similarities regarding perinatal PTSD symptomatology among different cultural backgrounds. Also, the GrTES-B’s five-factor structure can reveal significant information about the characteristics of Greek women who experience postpartum PTS symptoms.

The results of this study showed that GrTES-B demonstrated adequate internal consistency, since each of the five factors’ Cronbach’s α was greater than 0.7, indicating a reliable scale. This finding is consistent with results of previous studies [62,64]. The convergent validity findings were found to be acceptable, as there were observed significant positive correlations between almost all factors of the GrTES-B. The discriminant validity results revealed that the majority of the factors of the GrTES-B are correlated with a psychometric tool of depressive symptomatology. More specifically, the degree of correlation between the GrTES-B and the EPDS was found to be mainly at a low level, thus indicating the difference in conceptual content of the above instruments. In earlier studies, the TES-B was also administered in conjunction with a depression symptomatology scale [29,64].

The current study’s findings demonstrate that the GrTES-B has good and acceptable psychometric characteristics. The postpartum PTSD symptomatology among Greek women can be screened and evaluated efficiently via GrTES-B. This self-report tool’s simplicity and lack of complexity make it a potentially practical and affordable first screening tool for PTSD symptoms following childbirth in clinical settings. The GrTES-B can be administered by Greek midwifery care providers before a mental health professional performs a diagnostic evaluation. In addition, professionals working in midwifery settings in Greece may consider applying the GrTES-B for completion during women’s follow-up postpartum visits, given the questionnaire’s short completion time. Furthermore, the GrTES-B can be used either alone or in combination with formulated questions related to the childbirth experience. More specifically, professionals working in maternity care facilities can perform screening for postpartum PTSD symptomatology using specifically formulated questions that are mentioned in the literature focused on postpartum PTSD. Therefore, the first question they can ask a woman is the rather straightforward one, “How did you experience the delivery?” In the event that the woman had a negative experience, professionals may ask follow-up questions such as, “Would you describe your childbirth as traumatic or very upsetting?” or “Are you currently experiencing psychological symptoms that you think are caused by childbirth?” [76,77]. Following the aforementioned question formulation, suitable and validated screening questionnaires can be used. However, some implementation challenges with the completion of the validated tool may appear during the screening process. For instance, the postpartum mental health screening involves women’s propensity to underreport their symptoms [78]. Screening is hindered by a lack of awareness, feelings of shame, and stigma [79,80]. This implies that low introspective skills and desirability bias in reporting may limit the ability to identify postpartum PTSD symptoms based mainly on women’s symptom self-reporting. An additional challenge for Greek maternal care professionals is the fact that GrTES-B currently does not address all postpartum women; it is limited to those after a low-risk pregnancy.

Considering that early signs of PTSD may go undetected in postpartum women [31,51], that low attendance of follow-up postpartum visits is observed [81], and that the early identification and treatment of PTSD and its symptomatology is quite beneficial [52,82], the use of valid and reliable tools further strengthens healthcare professionals’ efforts to address this mental health issue. Also, the use of validated instruments can be combined with the implementation of interventions focused on postpartum PTSD. Results from recent systematic reviews regarding the efficacy of various interventions have been encouraging. Interventions such as expressive writing, eye-movement desensitization and reprocessing (EMDR), trauma-focused cognitive behavioral therapy (TFCBT), debriefing, and semi-structured midwife-led psychological counseling strategies were demonstrated to be effective in addressing postpartum PTSD. However, more studies are warranted to establish which interventions are most effective and acceptable for addressing postnatal PTSD and, furthermore, to establish clinical recommendations [83,84,85].

The present study has several limitations that should be mentioned. Although the current study’s findings show that the GrTES-B is a valid and reliable tool, clinical evaluation was not conducted in conjunction with the instrument’s validation. Also, the study’s sample consisted of postpartum women after a low-risk pregnancy and with regular prenatal care. Consequently, the findings may not apply to postpartum women after a high-risk pregnancy and with nontypical prenatal visits. Furthermore, since the sample was drawn from Greece’s capital, it was relatively homogeneous. A further study with a focus on the psychometric properties of GrTES-B in the population of postpartum women after high-risk pregnancy is suggested, since the factor analysis may differ in this group of women. In future investigations, it might be considered to include women with a disadvantageous background, i.e., low-income or minority groups, since they are at increased risk for traumatization [86]. Psychometric testing, alongside clinical interviews, is another recommendation for future studies with the GrTES-B. Furthermore, subsequent studies need to assess the GrTES-B’s long-term stability.

## 5. Conclusions

The Greek version B of the TES-B scale, i.e., GrTES-B, was found to be an effective screening tool for postpartum PTSD symptomatology in women after low-risk pregnancies in Greece. The GrTES-B’s psychometric properties indicate that it can be used as a screening instrument to identify postpartum PTSD symptomatology in a valid and reliable way, and therefore its integration into maternal and child health care could be considered. This integration could enhance early detection and intervention strategies, ultimately improving mental health outcomes for mothers.

## Figures and Tables

**Table 1:** Sample characteristics.

	N (%)
Nationality	
Greek	192 (96.0)
Other	8 (4)
Married/Living with partner	199 (99.5)
Occupation	
Employed	158 (79)
Unemployed	28 (14.0)
Household	14 (7.0)
Visited a specialist for psychological problems in the past	65 (32.5)
Psychotherapy in the past	45 (22.5)
Being abused during childhood	49 (24.5)
Being abused during adulthood	49 (24.5)
Stressful event during last year	82 (41.0)
Primigravida	104 (52.0)
Vaginal delivery	159 (80)
Caesarean section	40 (20.0)
Description of current childbirth experience	
Very positive	66 (33.0)
Mainly positive	107 (53.5)
Very negative	11 (5.5)
Mainly negative	16 (8.0)

**Table 2:** Descriptive statistics of response distribution for GrTES-B.

GrTES-Β	Never/Not at All	Rarely	Sometimes	Often
Item	Ν (%)	Ν (%)	Ν (%)	Ν (%)
a	10 (5.2)	62 (32.5)	69 (36.1)	50 (26.2)
b	91 (47.6)	74 (38.7)	19 (9.9)	7 (3.7)
c	122 (63.5)	49 (25.5)	14 (7.3)	7 (3.6)
d	76 (39.6)	76 (39.6)	25 (13)	15 (7.8)
1	144 (72)	32 (16)	19 (9.5)	5 (2.5)
2	195 (97.5)	5 (2.5)	0 (0)	0 (0)
3	189 (94.5)	7 (3.5)	4 (2)	0 (0)
4	149 (74.5)	29 (14.5)	17 (8.5)	5 (2.5)
5	172 (86)	21 (10.5)	6 (3)	1 (0.5)
6	163 (81.5)	25 (12.5)	5 (2.5)	7 (3.5)
7	168 (84)	22 (11)	7 (3.5)	3 (1.5)
8	125 (62.5)	49 (24.5)	25 (12.5)	1 (0.5)
9	127 (63.5)	38 (19)	28 (14)	7 (3.5)
10	141 (70.5)	24 (12)	29 (14.5)	6 (3)
11	170 (85)	22 (11)	7 (3.5)	1 (0.5)
12	181 (90.5)	14 (7)	5 (2.5)	0 (0)
13	181 (90.5)	14 (7)	5 (2.5)	0 (0)
14	98 (49)	59 (29.5)	34 (17)	9 (4.5)
15	90 (45)	65 (32.5)	39 (19.5)	6 (3)
16	79 (39.5)	52 (26)	54 (27)	15 (7.5)
17	85 (42.5)	64 (32)	41 (20.5)	10 (5)

**Table 3:** Mean scores of the GrTES-B, item–total correlations, and Cronbach’s a.

Factor	Item	Mean (SD)	Corrected Item–Total Correlation	Cronbach’s Alpha if Item Deleted	Cronbach’s Alpha (95% CI)
Anticipation of trauma	a		0.39	0.67	0.73
	b		0.31	0.72	(0.63–0.81)
	c	7.91 (2.31)	0.40	0.66	
	d		0.52	0.56	
Intrusion	1		0.56	0.58	0.73
	2		0.34	0.72	(0.65–0.82)
	3	6.1 (1.83)	0.48	0.64	
	4		0.70	0.48	
	5		0.43	0.64	
Avoidance	6		0.41	0.68	0.72
	7	4.02 (1.43)	0.36	0.68	(0.65–0.82)
	8		0.34	0.58	
Resignation	9		0.32	0.73	0.71
	10		0.56	0.60	(0.61–0.79)
	11	5.39 (1.86)	0.47	0.52	
	12		0.37	0.59	
Hyperstimulation	13		0.38	0.84	0.80
	14		0.68	0.72	(0.75–0.84)
	15	7.48 (2.99)	0.65	0.73	
	16		0.67	0.73	
	17		0.69	0.72	

**Table 4:** Correlations between the five factors of the GrTES-B.

		Intrusion	Avoidance	Resignation	Hyperstimulation
Anticipation of trauma	r	0.48	0.36	0.21	0.30
	*p*	<0.001	<0.001	0.003	<0.001
Intrusion	r	1.00	0.51	0.27	0.26
	*p*		<0.001	<0.001	<0.001
Avoidance	r		1.00	0.34	0.16
	*p*			<0.001	0.021
Resignation	r			1.00	0.43
	*p*				<0.001

**Table 5:** Correlations between the GrTES-B’s factors and EPDS.

		EPDS
Anticipation of trauma	r	0.37
	*p*	<0.001
Intrusion	r	0.33
	*p*	<0.001
Avoidance	r	0.06
	*p*	0.440
Resignation	r	0.33
	*p*	<0.001
Hyperstimulation	r	0.54
	*p*	<0.001

**Table 6:** Correlations between the GrTES-A and the GrTES-B.

		Anticipation of Trauma (A)	Intrusion (A)	Avoidance (A)	Resignation (A)	Hyperstimulation (A)
Anticipation of trauma (Β)	r	0.40	0.32	0.24	0.23	0.24
	*p*	<0.001	<0.001	0.001	0.001	0.001
Intrusion (Β)	r	0.34	0.43	0.30	0.31	0.36
	*p*	<0.001	<0.001	<0.001	<0.001	<0.001
Avoidance (Β)	r	0.23	0.20	0.24	0.19	0.12
	*p*	0.001	0.005	0.001	0.006	0.095
Resignation (Β)	r	0.14	0.22	0.21	0.27	0.21
	*p*	0.043	0.002	0.003	<0.001	0.002
Hyperstimulation (Β)	r	0.26	0.35	0.27	0.35	0.49
	*p*	<0.001	<0.001	<0.001	<0.001	<0.001

## Data Availability

The datasets analyzed in the current study are available from the corresponding author on reasonable request.

## Abbreviations

The following abbreviations are used in this manuscript:

CFA	Confirmatory factor analysis
CFI	Comparative fit index
DSM	Diagnostic and statistical manual of mental disorders
EPDS	Edinburgh postpartum depression scale
ETISR-SF	Early Trauma Inventory-Self Report-Short Form
GrTES	Greek version of the TES
IES-R	Impact of Event Scale—Revised
PCL-5	Posttraumatic Check List-5
PCL-C	PTSD Checklist—civilian scale
PTSD	Post-traumatic stress disorder
PTS	Post-traumatic stress
r	Spearman’s correlation coefficient
RMSEA	Root mean square error of approximation
TES	Traumatic event scale
TLI	Tucker–Lewis index
